# The *c-MET* Network as Novel Prognostic Marker for Predicting Bladder Cancer Patients with an Increased Risk of Developing Aggressive Disease

**DOI:** 10.1371/journal.pone.0134552

**Published:** 2015-07-30

**Authors:** Young-Won Kim, Seok Joong Yun, Phildu Jeong, Seon-Kyu Kim, Seon-Young Kim, Chunri Yan, Sung Phil Seo, Sang Keun Lee, Jayoung Kim, Wun-Jae Kim

**Affiliations:** 1 Department of Urology, College of Medicine, Chungbuk National University, Cheongju, South Korea; 2 Medical Genomics Research Center, Korea Research Institute of Bioscience and Biotechnology, Daejeon, Korea; 3 Korean Bioinformation Center, Korea Research Institute of Bioscience and Biotechnology, Daejeon, Korea; 4 Department of Functional Genomics, University of Science and Technology, Daejeon, Korea; 5 Department of Surgery, Harvard Medical School, Boston, MA, United States of America; 6 Departments of Surgery and Biomedical Sciences, Cedars-Sinai Medical Center, Los Angeles, CA, United States of America; Istituto dei tumori Fondazione Pascale, ITALY

## Abstract

Previous studies have shown that *c-MET* is overexpressed in cases of aggressive bladder cancer (BCa). Identification of crosstalk between *c-MET* and other RTKs such as *AXL* and *PDGFR* suggest that *c-MET* network genes (*c-MET*-*AXL*-*PDGFR*) may be clinically relevant to BCa. Here, we examine whether expression of *c-MET* network genes can be used to identify BCa patients at increased risk of developing aggressive disease. *In vitro* analysis, *c-MET* knockdown suppressed cell proliferation, invasion, and migration, and increased sensitivity to cisplatin-induced apoptosis. In addition, *c-MET* network gene (*c-MET*, *AXL*, and *PDGFR*) expression allowed discrimination of BCa tissues from normal control tissues and appeared to predict poor disease progression in non-muscle invasive BCa patients and poor overall survival in muscle invasive BCa patients. These results suggest that *c-MET* network gene expression is a novel prognostic marker for predicting which BCa patients have an increased risk of developing aggressive disease. These genes might be a useful marker for co-targeting therapy, and are expected to play an important role in improving both response to treatment and survival of BCa patients.

## Introduction

Overexpression of receptor tyrosine kinases (RTKs) occurs in cases of aggressive bladder cancer (BCa); thus RTK-targeting therapies are recommended for such patients [1, 2]. Pharmacological inhibition of RTK activity (e.g., with gefitinib) is the gold standard treatment for BCa patients, although it has met with limited success [3, 4].

The *c-MET* proto-oncogene, which is located on chromosome 7q21-31 [5], is overexpressed in BCa. *c-MET* is activated by its ligand, hepatocyte growth factor (HGF), and induces increased proliferation, migration, motility, and invasion of BCa cells [6]. Upon stimulation and dimerization of *c-MET*, tyrosine phosphorylation occurs at specific sites within the intracellular domain (i.e., Y1234, Y1235, Y1349, and Y1356), which increases the intrinsic activity of tyrosine kinases and leads to the recruitment of many signaling proteins, including growth factor receptor-bound protein 2 (GRB2), Grb2-associated binder-1 (GAB1), Src homology 2 domain containing (SHC), phospholipase C1 (PLC1), and phosphoinositide 3-kinase (PI3-K) [7]. The Ras/Erk-MAPK, PI3-K/Akt/mTOR [8], and STAT3 signaling pathways are also activated, thereby inducing several biological responses [6, 9].

Overexpression of *c-MET* correlates with BCa metastasis [7, 10]; indeed, *c-MET* is overexpressed in more than 60% of locally advanced and metastatic BCa cases [5], and is linked to poor survival [11]. Considering that the dimerization of RTKs is important for controlling their biological function in the context of cancer, crosstalk between *c-MET* and other RTKs should be investigated carefully if we are to understand the role of *c-MET* in human cancer progression. Sections of primary tumor from patients with a rare type of BCa, called neuroendocrine (NE) BCa, show *c-MET* expression [12]. This suggests that NE BCa may be a suitable target for *c-MET* inhibitors. A previous study showed that a member of the *c-MET* family, recepteur d’origine Nantais (RON), forms a heterodimer with epidermal growth factor receptor (EGFR) [13]. In addition, RTK microarray analysis revealed that RTKs such as *AXL* and *PDGFR* crosstalk with *c-MET* [11]. *AXL* and *PDGFR* are associated with aggressive breast [14], kidney [15], lung [16, 17], and prostate cancers [18, 19], suggesting that *c-MET*-*AXL*-*PDGFR* may be clinically relevant to BCa [11].

The aim of the present study was to examine the clinical association between the expression of *c-MET* network genes (*c-MET*-*AXL*-*PDGFR*) and disease outcome for BCa patients, and to investigate whether *c-MET* network genes can be used to identify BCa patients at increased risk of developing aggressive disease.

## Materials and Methods

### Patients and tissue samples

Primary tumor samples from patients who underwent transurethral resection (TUR) or radical cystectomy at Chungbuk National University in South Korea were histologically verified as urothelial carcinoma. Normal bladder mucosa was harvested from patients with benign diseases such as benign prostatic hyperplasia (BPH), ureter stones, and stress urinary incontinence, after informed consent. All control tissues were histologically confirmed as normal. Patients with concomitant carcinoma *in situ* (CIS), CIS lesions alone, a short follow-up period (less than 6 months), or for whom data were incomplete, were excluded to yield a more homogeneous study population. A total of 165 (135 male and 30 female; average age, 65 years) BCa patients and 34 controls (19 male and 15 female; average age, 54 years) were enrolled. All tumors were macro-dissected (typically within 15 minutes of surgical resection), and each BCa specimen was confirmed by pathological analysis of a fresh frozen tissue section derived from TUR or cystectomy specimens. Tumor samples were then frozen in liquid nitrogen and stored at -80°C until use. NMIBC patients underwent a second TUR 2–4 weeks after initial resection if the BCa specimen did not include the proper muscle layer or when a high-grade tumor was detected. Patients with a T1 tumor, multiple tumors, large tumors (>3 cm in diameter), or high-grade Ta NMIBC received one cycle of intravesical treatment [bacillus Calmette-Guérin (BCG) or mitomycin-C]. Response to treatment was assessed by cystoscopy and urinary cytology. Patients who were disease-free within 3 months of treatment were followed-up every 3 months for the first 2 years and then every 6 months thereafter. MIBC patients with clinically localized or locally advanced tumors and good Eastern Cooperative Oncology Group (ECOG) performance status (0 or 1) underwent radical cystectomy and complete pelvic lymph node dissection. Patients not eligible for radical cystectomy due to metastatic disease, poor life expectancy, or poor ECOG performance status (≥2) underwent TUR or biopsy for histopathological diagnosis. Patients with pT3, pT4, or node-positive disease (based on the analysis of radical cystectomy specimens) and those with metastatic disease but good performance status received at least four cycles of cisplatin-based chemotherapy. Patients who refused or did not complete an imaging work-up [computed tomography (CT) scan or magnetic resonance imaging (MRI)] at least once every 3 months to evaluate responses were excluded from further analysis.

Tumors were staged and graded according to the 2002 TNM classification and the *European Association of Urology (EAU)* guidelines based on the 1973 *WHO* grading system [20, 21]. Recurrence was defined as recurrence of primary NMIBC with a lower or the same pathological stage, and progression was defined as the identification of T2 or higher stage disease upon relapse. In the case of MIBC, progression was defined as locoregional recurrence or a new distant metastasis in cystectomized patients and a ≥20% increase in the mass of the primary tumor or a new distant metastasis in non-cystectomized patients.

### RNA extraction and reverse transcription to cDNA

RNA was isolated from tissues by homogenization with 1 ml of TRIzol reagent (Invitrogen, Carlsbad, CA) in a 5 ml glass tube. The homogenate was then transferred to a 1.5 ml tube and mixed with 200 ml of chloroform. After incubating for 5 min at 4°C, the homogenate was centrifuged for 13 min at 13,000 g at 4°C. The upper aqueous phase was transferred to a clean tube containing 500 ml of isopropanol. The mixture was incubated for 60 min at 4°C and then centrifuged for 8 min at 13,000 g at 4°C. The upper aqueous phase was discarded and mixed with 500 ml of 75% ethanol and centrifuged for 5 min at 13,000 g at 4°C. The upper aqueous layer was discarded, and the pellet was dried at room temperature, dissolved in DEPC-treated water, and then stored at -80°C. The quality and integrity of the RNA were confirmed using a Nanodrop device. cDNA was prepared from 1 mg of total RNA using a First-Strand cDNA Synthesis Kit (Amersham Biosciences Europe GmbH, Freiburg, Germany) according to the manufacturer’s protocol.

### Cell culture and transfection

T24 BCa cells were obtained and cultured according to the instructions provide by the ATCC. Media were supplemented with 10% fetal bovine serum, 2% glutamine, and 1% antibiotics (Invitrogen, Carlsbad, CA), and cells were maintained under a humidified atmosphere of 5% CO2 at 37°C. For the knockdown experiments, cells were transiently transfected with 200 pmol siRNA pool to silence MET (MET siRNAs, Life Technologies, catalog number 103545, 103551, 103557, 103767 and 103769) or negative control siRNAs, using Lipofectamine 2000 (Invitrogen).

### Proliferation assay

SiRNA-transfected cells were seeded in 24-well plates at a density of 1 × 10^4^/well. Cells were then stained with crystal violet and counted 7 days later [22].

### Anchorage-independent soft agar growth assay

SiRNA-transfected cells (1 × 10^4^) were seeded into 3 ml of 0.35% agar in FBS-containing culture medium and overlaid onto 2 ml of 0.7% agar in FBS-containing culture medium in 6-well plates. Images of 3-(4, 5-dimethylthiaz-113 olyl-2)-2, 5-diphenyltetrazolium bromide (MTT)-stained colonies were captured under a Zeiss microscope as described previously [22].

### Invasion assay

Cells (3 × 10^5^ cells/ml) were counted and seeded onto collagen-coated inserts (Millipore Corp., Billerica, MA). After 16 h, the cells that migrated to the bottom surface of the inserts were stained with crystal violet solution. The dye was extracted from the cells using 10% acetic acid solution, and absorbance was read in a FLUOstar Omega microplate reader (BMG Labtech, Cary, NC) as previously described [22].

### Cell apoptosis assay

T24 cells transiently transfected with siRNA were incubated in medium with or without 10 μM cisplatin for 8 hours. Cell viability was measured in an MTT assay as previously described [23]. Cell apoptosis was quantified by measuring the metabolically active mass of the treated cells after normalization against untreated cells.

### Wound-healing (in vitro scratch) assay

T24 cells grown on poly-L-lysin were co-transfected with the plasmid encoding GFP. They were then subjected to *in vitro* scratch assay with images captured at 0 and 16 h after incubation using fluorescence microscope. Cells moved from the edge of the scratch toward the center of the scratch (marked by yellow dotted lines).

### Western blot analysis

Transfected T24 cells were quickly harvested, flash frozen in liquid nitrogen, and stored at -80°C. Total protein was extracted in lysis buffer [1% Nonidet P-40, 50 mM Tris (pH 7.4), 10 mM NaCl, 1 mM NaF, 5 mM MgCl2, 0.1 mM EDTA, 1 mM phenylmethylsulfonyl fluoride, and Complete protease inhibitor cocktail tablet (Roche Diagnostics GmbH, Mannheim, Germany)] at the indicated conditions and centrifuged at 12,500 *g* for 15 min. 25μg proteins per each conditions were subjected to SDS-PAGE gel running, which were transferred to nitrocellulose membranes for Western blot analysis. After blocking with 10% BSA/PBST for 1h, membranes were incubated with specific antibodies against *c-MET*, MMP2, MMP9 or β-actin. The blots were visualized by enhanced chemiluminescence.

### Computational analysis

To study the association between *c-MET* network genes and clinical parameters in BCa patients, we examined the expression profiles of these genes in BCa patients using previously obtained microarray data (accession number GSE13507). Microarray data were available for 165 patients. Clinical outcomes, including progression-free survival (PFS), overall survival (OS), and cancer-specific survival (CSS) were obtained from medical records. The correlation between gene expression and disease outcome was examined using Cox proportional hazards regression analysis. Kaplan–Meier (KM) survival curve analysis using a subset of gene expression profiles from patients with “low” and “high” expression of each gene were used to identify the effects of *c-MET* network gene expression on BCa. The 50^th^ percentile (median) of gene expression was used as the cuff-off value. The log-rank test was performed to assess the significance of differences between two survival curves.

### Ethical statement

The study was approved by the Ethics Committee of Chungbuk National University. All subjects provided written informed consent. Sample collection and analysis were approved by The Institutional Review Board of Chungbuk National University.

## Results

### Clinical and pathological characteristics of BCa patients

The mean age of the 165 patients in the study cohort was 65.2 ± 12.0 years, and the mean follow-up period was 48.4 months. Of the 165 patients, 62.4% (103/165) had NMIBC and 37.6% (62/165) had MIBC. The mean age of the 34 patients in the normal control cohort was 54.0 ± 10.4 years. The baseline characteristics of the patients and controls are presented in Table 1.

**Table 1:** Clinico-pathological features of patients with bladder cancer and normal controls.

Varibles	Study cohort (n = 165)	NC (n = 34)
Age—yr (mean)	65.2 ± 12.0	54 ± 10.4
Gender—no. of patients (%)		
Male	135 (81.8)	19 (55.9)
Female	30 (18.2)	15 (44.1)
Grade—no. of patients (%)		
Low	105 (63.6)	
High	60 (36.4)	
Stage—no. of patients (%)		
NMIBC	103 (62.4)	
Ta	23 (22.3)	
T1	80 (77.7)	
MIBC	62 (37.6)	
T2N0M0	26 (41.9)	
T3N0M0	13 (21.0)	
T4/Any T N+/M+	23 (37.1)	
Recurrence—no. of patients with NMIBC (%)		
No	67 (65.0)	
Yes	36 (35.0)	
Progression—no. of patients (%)		
NMIBC		
No	92 (89.3)	
Yes	11 (10.7)	
MIBC		
No	42 (67.7)	
Yes	20 (32.3)	
Survival—no. of patients with MIBC (%)		
Cancer-specific		
Alive	33 (53.2)	
Deceased	29 (46.8)	
Overall survival		
Alive	28 (45.2)	
Deceased	34 (54.8)	
Mean follow-up—months	48.4	

Abbreviations: NMIBC, non-muscle invasive bladder cancer; MIBC, muscle invasive bladder cancer; NC, normal control.

### Loss of *c-MET* suppresses BCa cell proliferation and invasion and increases sensitivity to cisplatin-induced apoptosis

**Fig 1:**
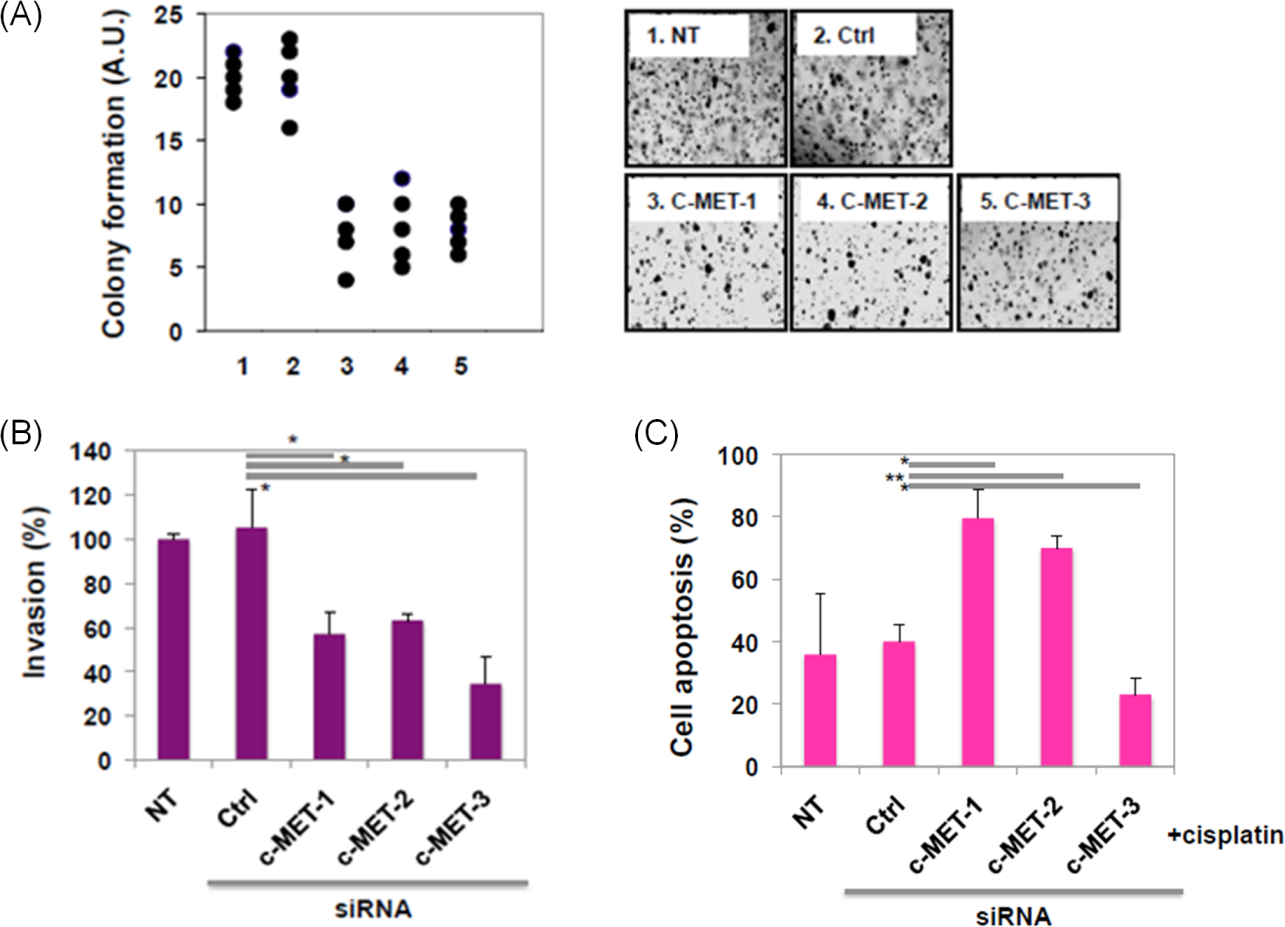
Loss of *c-MET* reduced anchorage-independent proliferation (A) and invasion (B) of T24 bladder cancer cells with an increased cisplatin-induced cell apoptosis (C). All experiments were performed using three *c-MET* knockdown cell lines (si*c-MET*-1, si*c-MET*-2, and si*c-MET*-3) transfected with different *MET*siRNAs, and two controls cell lines (Ctrl and NT). Ctrl, control; NT, non-transfected.*p<0.05.

Previous studies suggest that stromal HGF signaling via the *c-MET* pathway increases invasion and metastasis of BCa cells [6, 11, 13]; therefore, we sought to determine the effects of *c-MET* silencing on proliferation and invasion, and on the apoptotic response to cisplatin (a major chemotherapeutic agent used to treat BCa patients). We found that the BCa cells in which *c-MET* was knocked down formed fewer (and smaller) colonies than the negative control cells, suggesting a reduction in cell proliferation in soft agar (Fig 1A). The cell invasion assay showed that BCa cells harboring intact *c-MET* were more invasive than those in which *c-MET* was knocked down. *c-MET*-silenced T24 cells were much less invasive than controls cells (non-transfected cells and cells transfected with control siRNA) (Fig 1B). We next examined the consequence of *c-MET* loss on cell apoptosis in an MTT assay. *c-MET* knockdown cells showed increased sensitivity to cisplatin-induced apoptosis (Fig 1C).

### Loss of *c-MET* inhibits the cell migration of BCa cells by downregulating MMP2 and MMP9

**Fig 2:**
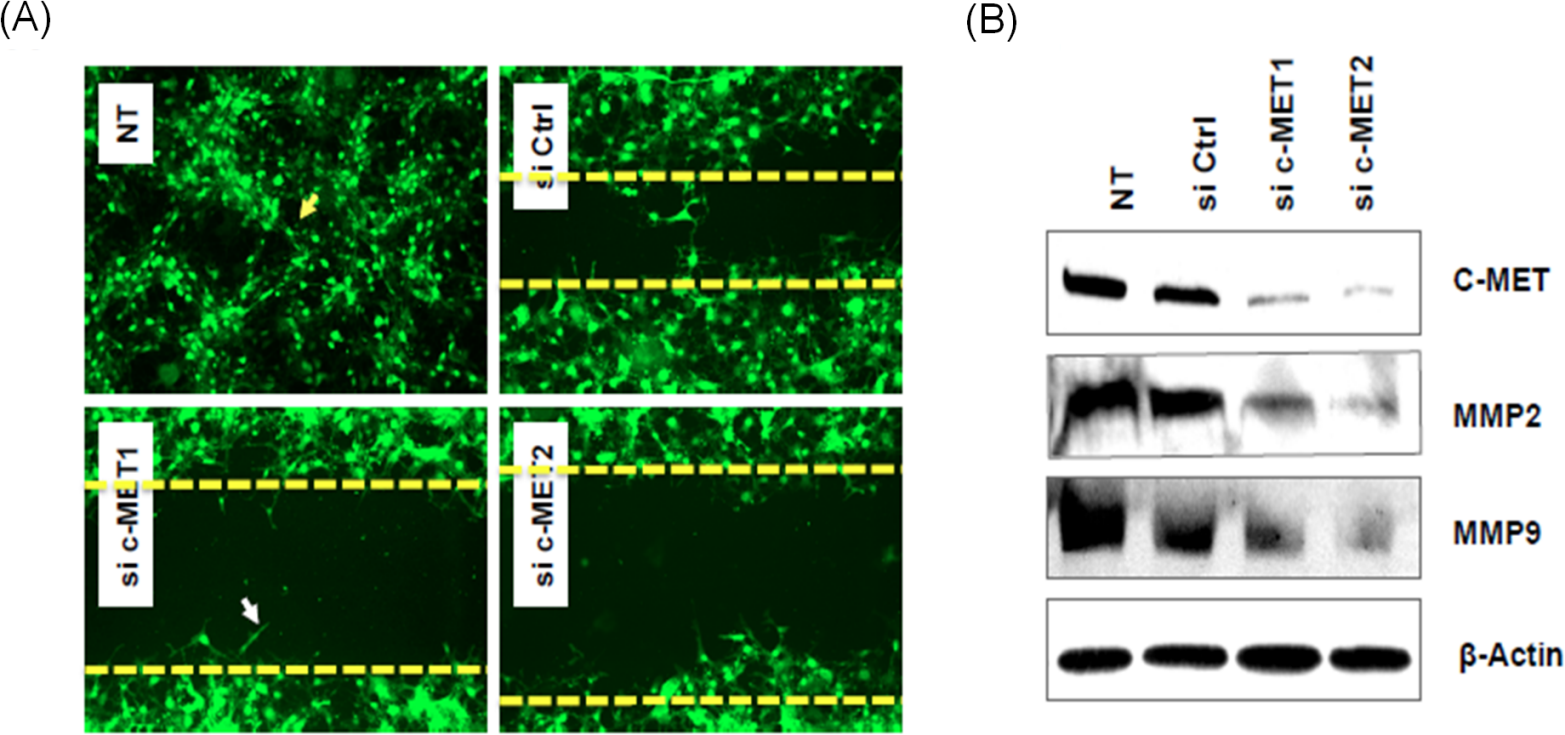
MMP2 and MMP9 may be downstream effectors of *c-MET* knockdown, leading to suppression of migration in T24 bladder cancer cells. (A) Wound-healing assay showing that knockdown of c-MET inhibitsthe migration of T24 cells. (B) Loss of *c-MET* downregulated the expression of matrix metalloproteinases (MMP)-2 and MMP-9. All experiments were performed using two *c-MET* knockdown cell lines (si*c-MET*-1 and si*c-MET*-2) transfected with different MET siRNAs, and two control cell lines (Ctrl and NT). Ctrl, control; NT, non-transfected.

We examined cell migration and MMP2 and MMP9 expression in BCa cells. Wound-healing assay (also called as in vitro scratch assay) showed that *c-MET*-knockdown cells migrated less efficiently than control cells (Fig 2A). We also found that knocking down *c-MET* downregulated the expression of MMP2 and MMP9 in BCa cells (Fig 2B).

### Expression of *c-MET* correlates with OS in MIBC patients

**Fig 3:**
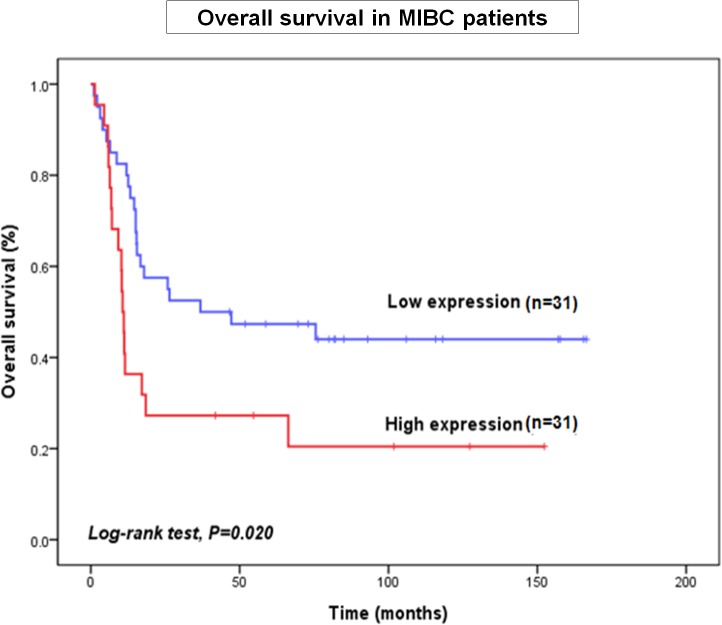
Kaplan–Meier curves showing that high expression of *c-MET* genes correlates with poor overall survival of MIBC patients.

To answer the question of whether *c-MET* network genes are involved in BCa progression and aggressiveness, we analyzed the expression of mRNA for these genes in a DNA microarray and compared the results with disease characteristics such as tumor grade (G), tumor stage (T, N, and M), tumor size, recurrence, progression, and CSS. Further comparisons were then performed after patients were categorized into NMIBC and MIBC groups. The results revealed that *c-MET* mRNA expression in MIBC patients correlated significantly with OS (p = 0.023; HR, 2.107; 95% confidence interval (CI), 1.110–3.998) (Table 2). These data were confirmed by KM survival curve analysis (Fig 3). BCa patients with high levels of *c-MET* expression showed poorer survival than those with low expression (log-rank test, p = 0.020).

**Table 2:** *c-MET* gene expression correlates to OS of MIBC patients.

	Gene symbols	p-value	Hazard ratio	95% confidence interval
**NMIBC recurrence**	***c-MET***	**0.120**	**1.640**	**0.879–3.060**
**NMIBC progression**	***c-MET***	**0.363**	**1.682**	**0.549–5.154**
**MIBC progression**	***c-MET***	**0.237**	**1.662**	**0.716–3.858**
**MIBC CSS**	***c-MET***	**0.142**	**1.728**	**0.832–3.590**
**MIBC OS**	***c-MET***	**0.023**	**2.107**	**1.110–3.998**

NMIBC, non-muscle invasive bladder cancer; MIBC, muscle invasive bladder cancer; CSS, cancer-specific survival; OS, overall survival.

### The expression of *AXL* can distinguish between NMIBC and MIBC patients and healthy controls

We next examined the clinical association between known *c-MET* partners, *AXL* and *PDGFR*, and BCa progression. The results showed that *AXL* expression clearly correlated with both NMIBC and MIBC. Bladder tumors (NMIBC and MIBC) showed 0.471-fold higher expression of *AXL* mRNA than control tissues. *AXL* mRNA expression by NMIBC (p < 0.0001, false discovery rate (FDR) < 0.0001) and MIBC (p = 0.0001, FDR = 0.0006) was significantly higher than that in normal controls (Table 3). *AXL* mRNA expression in NMIBC tissue was approximately 1.532-fold higher than that in MIBC tissue (Table 3).

**Table 3:** *AXL* mRNA expression in bladder cancer (NMIBC and MIBC) patients and normal controls.

	Gene symbols	p-value	FDR	Fold change
**Normal vs. BT (NMIBC+ MIBC)**	***AXL***	**<0.0001**	**<0.0001**	**0.471**
**Normal vs. NMIBC**	***AXL***	**<0.0001**	**<0.0001**	**0.401**
**Normal vs. MIBC**	***AXL***	**0.0001**	**0.0006**	**0.614**
**NMIBC vs. MIBC**	***AXL***	**<0.0001**	**0.0008**	**1.532**

FDR, false discovery rate; NMIBC, non-muscle invasive bladder cancer; MIBC, muscle invasive bladder cancer; BT, bladder tumor

### Expression of *PDGFR* isoforms is significantly altered in BCa, and high expression of *PDGFRL* predicts poor survival

To test whether *PDGFR* is useful as a diagnostic classifier, we examined the expression of three different isoforms (i.e., *PDGFRA*, *PDGFRB*, and *PDGFRL*). We found that the expression of *PDGFR* isoforms could be used to distinguish NMIBC and MIBC samples from normal controls. The expression of *PDGFRA* mRNA clearly discriminated bladder tumors (NMIBC and MIBC) from normal control tissues (p < 0.0001, FDR < 0.0001), with *PDGFRA* expression in NMIBC and MIBC being approximately 0.274-fold higher than that in controls. The expression of *PDGFRB* was also clearly different between tumors and normal tissues (p = 0.0001, FDR < 0.0001). *PDGFRB* expression in NMIBC was significantly greater than that in normal controls (p < 0.0001), but no significant difference was shown between MIBC and normal controls (p = 0.0698). Similarly, *PDGFRL* was differentially expressed in NMIBC and normal controls, with a modest increase (0.804-fold) (p < 0.0001) in the former. Thus, it is likely that *PDGFR* expression is increased in all types of BCa. It is noteworthy that the expression of *PDGFR* isoforms in tissues from NMIBC patients was generally higher than that in tissues from MIBC patients (Table 4).

**Table 4:** Expression of *PDGFR* isoforms in bladder cancer (NMIBC and MIBC) patients.

	Gene symbols	p-value	FDR	Fold change
**Normal vs. BT (NMIBC+ MIBC)**	***PDGFRA***	**<0.0001**	**<0.0001**	**0.274**
**Normal vs. NMIBC**	***PDGFRA***	**<0.0001**	**<0.0001**	**0.256**
**Normal vs. MIBC**	***PDGFRA***	**<0.0001**	**<0.0001**	**0.308**
**NMIBC vs. MIBC**	***PDGFRA***	**0.0922**	**0.2335**	**1.205**
**Normal vs. BT (NMIBC+ MIBC)**	***PDGFRB***	**<0.0001**	**<0.0001**	**0.569**
**Normal vs. NMIBC**	***PDGFRB***	**<0.0001**	**<0.0001**	**0.465**
**Normal vs. MIBC**	***PDGFRB***	**0.0698**	**0.1354**	**0.796**
**NMIBC vs. MIBC**	***PDGFRB***	**<0.0001**	**0.0001**	**1.712**
**Normal vs. BT (NMIBC+ MIBC)**	***PDGFRL***	**0.2057**	**0.3011**	**0.921**
**Normal vs. NMIBC**	***PDGFRL***	**0.0001**	**0.0006**	**0.804**
**Normal vs. MIBC**	***PDGFRL***	**0.0835**	**0.1562**	**1.154**
**NMIBC vs. MIBC**	***PDGFRL***	**<0.0001**	**<0.0001**	**1.435**

FDR, false discovery rate; NMIBC, non-muscle invasive bladder cancer; MIBC, muscle invasive bladder cancer; BT, bladder tumor

**Fig 4:**
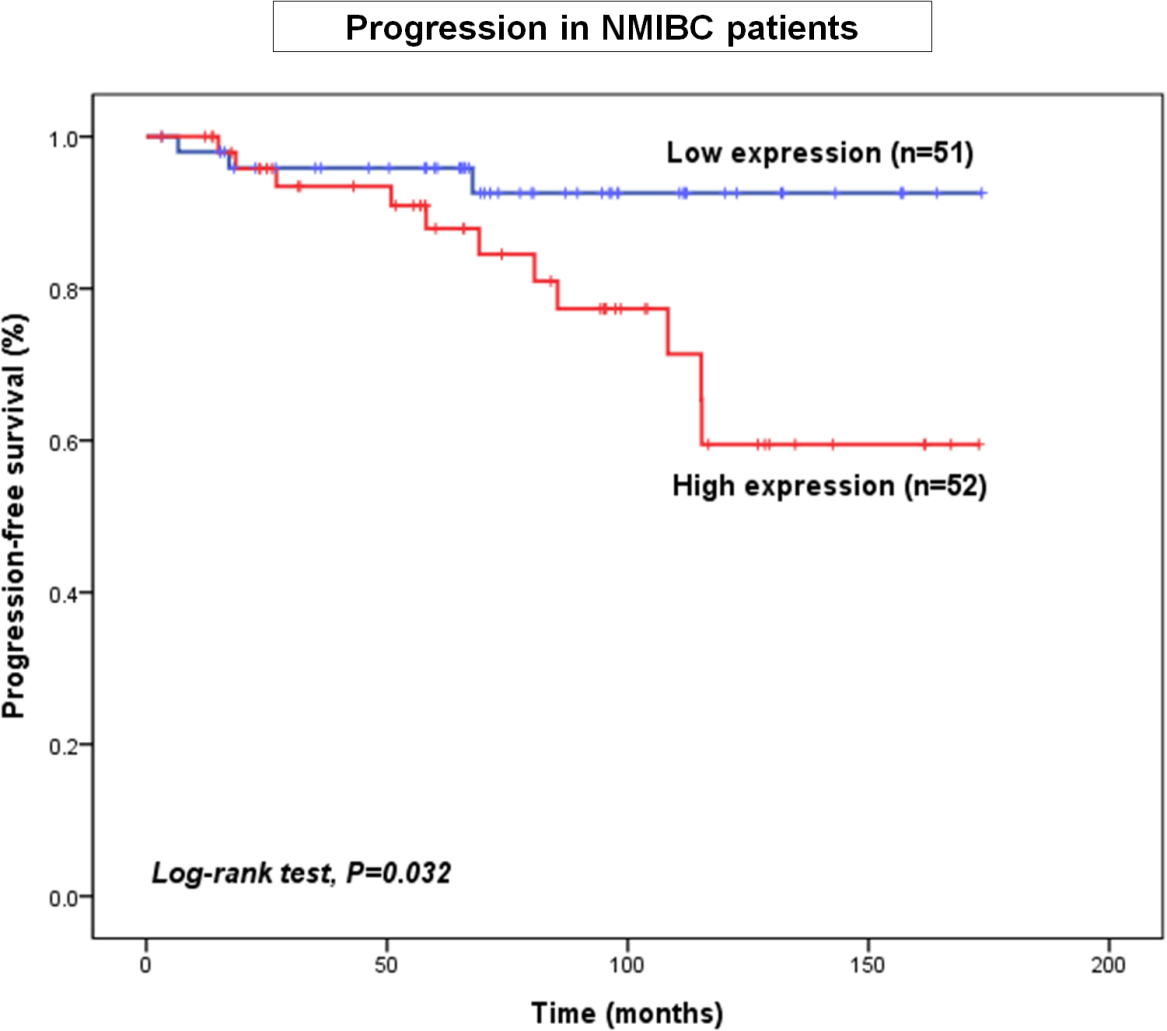
Kaplan–Meier curves showing that high expression of *PDGFRL* (one of the *PDGFR* isoforms) correlates with disease progression in NMIBC patients.

Next, to understand the clinical relevance of increased *PDGFR* expression in BCa, we examined the clinical correlation between *PDGFR* isoform expression and disease progression. We found that *PDGFRL* expression was significantly correlated with NMIBC progression (p = 0.046; HR, 3.675; 95% CI, 1.024–13.188) (Table 5). KM survival analysis showed that NMIBC patients with high expression of *PDGFRL* showed poorer PFS than those with low expression of *PDGFRL* (log-rank test, p = 0.032) (Fig 4).

**Table 5:** Expression of *PDGFR* isoforms and clinicopathological features of bladder cancer.

	Gene symbols	p-value	Hazard ratio	95% confidence interval
**NMIBC recurrence**	***PDGFRA***	**0.614**	**1.173**	**0.630–2.184**
**NMIBC progression**	***PDGFRA***	**0.466**	**0.660**	**0.215–2.021**
**MIBC progression**	***PDGFRA***	**0.941**	**1.032**	**0.445–2.397**
**MIBC CSS**	***PDGFRA***	**0.410**	**1.373**	**0.645–2.923**
**MIBC OS**	***PDGFRA***	**0.603**	**1.190**	**0.617–2.294**
**NMIBC recurrence**	***PDGFRB***	**0.551**	**0.826**	**0.441–1.547**
**NMIBC progression**	***PDGFRB***	**0.981**	**1.014**	**0.339–3.031**
**MIBC progression**	***PDGFRB***	**0.796**	**1.153**	**0.391–3.403**
**MIBC CSS**	***PDGFRB***	**0.681**	**0.837**	**0.359–1.951**
**MIBC OS**	***PDGFRB***	**0.873**	**0.939**	**0.430–2.046**
**NMIBC recurrence**	***PDGFRL***	**0.295**	**1.398**	**0.746–2.621**
**NMIBC progression**	***PDGFRL***	**0.046**	**3.675**	**1.024–13.188**
**MIBC progression**	***PDGFRL***	**0.283**	**1.945**	**0.577–6.561**
**MIBC CSS**	***PDGFRL***	**0.894**	**0.944**	**0.406–2.195**
**MIBC OS**	***PDGFRL***	**0.861**	**0.935**	**0.444–1.972**

NMIBC, non-muscle invasive bladder cancer; MIBC, muscle invasive bladder cancer; CSS, cancer-specific survival; OS, overall survival.

### Expression levels of *c-MET* network genes is significantly correlated with disease progression in NMIBC patients and with OS in MIBC patients

**Fig 5:**
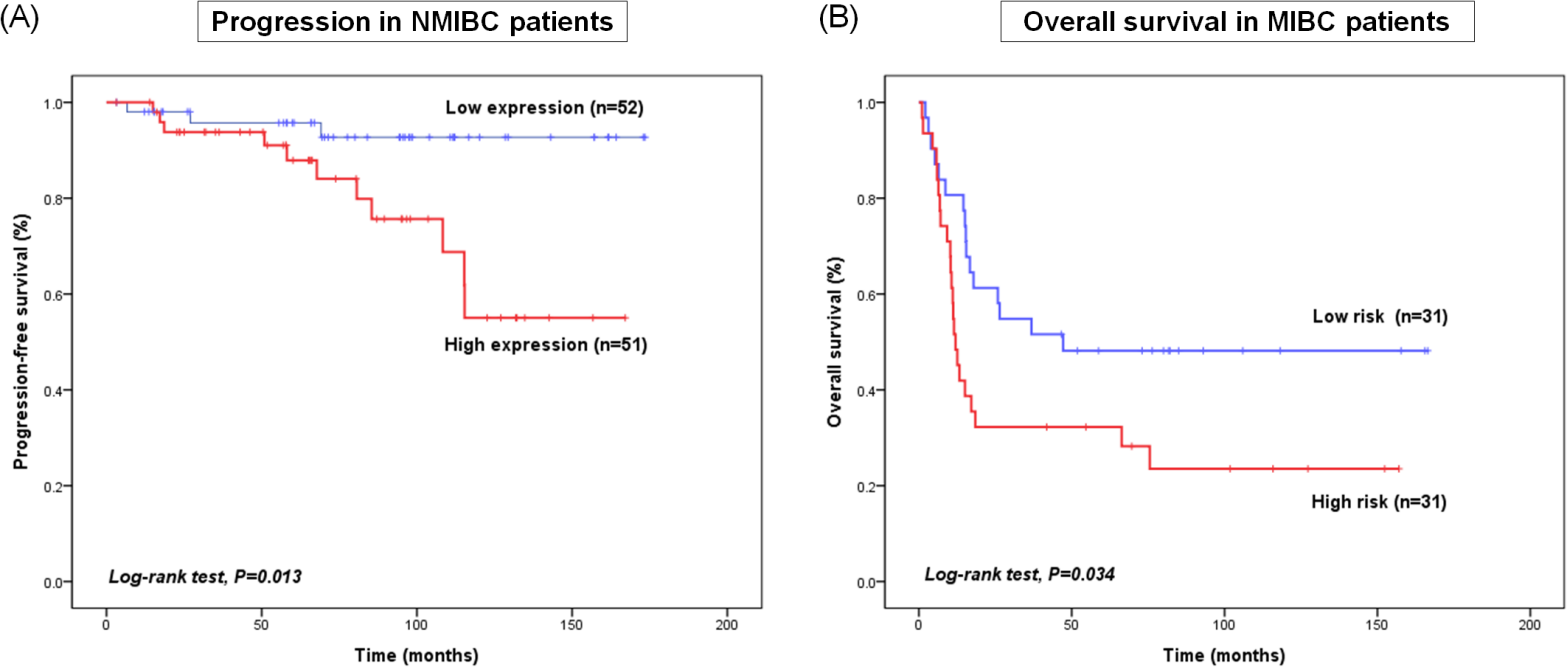
Kaplan–Meier curves showing that high expression of *c-MET* network genes correlates with (A) poor progression-free survival in NMIBC patientsand (B) poor overall survival in MIBC patients.

To identify the clinical importance of *c-MET* network genes, we examined the association between *c-MET* network gene expression (*c-MET*, *AXL*, and *PDGFR*) and BCa prognosis. Expression of *c-MET* network genes was based on an assessment of the risk score for each patient calculated by combining the expression levels of all three genes. We found that expression of *c-MET* network genes correlated significantly with disease progression in NMIBC patients (p = 0.023; HR, 4.386; 95% CI, 1.221–15.757) and with OS in MIBC patients (p = 0.038; HR, 1.976; 95% CI, 1.039–3.759) (Table 6). KM survival analysis showed that NMIBC patients with high expression of *c-MET* network genes showed poorer PFS (log-rank test, p = 0.013) than those with low expression. Similarly, MIBC patients (log-rank test, p = 0.034) with high expression of c-MET network genes showed poorer OS than those with low expression (Fig 5).

**Table 6:** *c-MET* network gene expression correlates with NMIBC progression and with OS of MIBC patients.

	Gene symbols	p-value	Hazard ratio	95% Confidence interval
**NMIBC recurrence**	***c-MET network***	**0.143**	**1.600**	**0.853–3.002**
**NMIBC progression**	***c-MET network***	**0.023**	**4.386**	**1.221–15.757**
**MIBC progression**	***c-MET network***	**0.499**	**1.328**	**0.584–3.016**
**MIBC CSS**	***c-MET network***	**0.348**	**1.403**	**0.691–2.848**
**MIBC OS**	***c-MET network***	**0.038**	**1.976**	**1.039–3.759**

NMIBC, non-muscle invasive bladder cancer; MIBC, muscle invasive bladder cancer; CSS, cancer-specific survival; OS, overall survival.

## Discussion

The results of the present study suggest that loss of *c-MET* makes BCa cells less invasive and more susceptible to cisplatin. Also, the expression pattern of *c-MET* network genes allows the discrimination of BCa tissues from normal control tissues and appears to predict poor clinical outcomes in a Korean patient population.

Aberrant *c-MET* expression occurs in various cancers and is associated with a poor prognosis [24]. Overexpression of *c-MET* in BCa is associated with poor OS and metastasis-free survival [5, 7, 10]. Yeh et al. reported that overexpression of *c-MET* is positively associated with muscle invasion and poor long-term survival (p < 0.001) [11]. Previous reports suggest that *c-MET* expression is closely associated with both tumor aggressiveness and patient survival [5, 7, 10, 11, 18]. The results presented herein are in agreement with those in previous studies. We found that overexpression of *c-MET* was significantly associated with poor survival, particularly OS in MIBC patients. This suggests that inhibiting *c-MET* expression may play an important role in preventing BCa progression and improve patient survival.

*In vitro* analysis showed that *c-MET* knockdown suppressed cell proliferation, invasion, and migration, and reduced the expression of MMP2 and MMP9. This was accompanied by increased sensitivity to cisplatin-induced apoptosis. MMP2 and MMP9 degrade extracellular matrix proteins, thereby facilitating cell invasion and metastasis [25, 26]. These results indicate that *c-MET* inhibition is likely to reduce cancer invasion and metastasis and to improve the survival of cancer patients. Thus, a more focused understanding of the importance of the *c-MET* inhibition is needed if we are to develop inhibitors that target *c-MET* in various tumors. Recently, several *c-MET*-targeting drugs were tested in clinical trials, and all show promising clinical activity with acceptable side effects [27]. For example, tivantinib (also called ARQ197) and a dual inhibitor of *c-MET*/VEGFR2 (foretinib) were studied in phase I to II clinical trials in patients with papillary renal cell carcinoma and advanced hepatocellular carcinoma, respectively [24]. A novel multikinase inhibitor of *MET*, *VERFR1*, *AXL*, *TIE2*, *KIT*, *FLT3*, and *RET*, called cabozantinib (also known as XL184), inhibits the growth, metastasis, and angiogenesis in pancreatic cancer and glioblastoma, and reduces resistance to gemcitabine. In particular, clinical trials in metastatic castration-resistant prostate cancer (mCRPC) patients reported a promising effect on PFS, bone metastasis, and pain [28]. However, tumors that initially show a good response to MET inhibitors may later develop resistance [24]. Acquired resistance to MET inhibitors develops via multiple mechanisms including genetic alterations (e.g., secondary *EGFR* T790M mutation), MET amplification, and activated signaling pathways [29]. Thus, multiple combination target therapy or co-targeting therapy might be necessary to prevent drug resistance and to achieve beneficial outcomes [24].

It is important to examine the crosstalk between *c-MET* and other RTKs because crosstalk partners of *c-MET* may be important biomarkers for co-targeting therapy and help to prevent resistance to individual MET inhibitors. *RON*, *AXL* and *PDGFR* have a crosstalk with *c-MET*. Overexpression of *AXL* and *PDGFR* is associated with aggressiveness and prognosis of a tumor series [14–19]. The results presented herein are consistent with previous studies in this respect; however, there were some differences. In contrast to the study by Yet et al., which evaluated the association between *PDGFRA* and BCa progression, we examined all three *PDGFR* isoforms: *PDGFRA*, *PDGFRB*, and *PDGFRL*. However, only *PDGFRL* was associated with NMIBC progression. Most studies that aimed to identify a correlation between BCa progression and *PDGFR* examined the *PDGFRA* and *PDGFRB* isoforms. Therefore, the present study is the first to identify a significant association between *PDGFRL* expression and BCa prognosis. In addition, Yet et al. only examined the roles of *AXL* and *PDGFR* in advanced cases [11]. Here, we showed that expression of *AXL* and *PDGFR* distinguished NMIBC and MIBC from healthy controls. In particular, expression of both of these genes was higher in NMIBC patients than in MIBC patients. Thus, *AXL* and *PDGFRL* may be more specific for NMIBC than MIBC. A large validation is needed to clarify the roles and effects of *PDGFR* isoforms on BCa (NMIBC and MIBC) prognosis.

We also found that *c-MET* network gene (*c-MET*, *AXL*, and *PDGFR*) expression was closely associated with disease progression in NMIBC patients and with poor survival (especially OS) in MIBC patients. These results suggest that inhibiting the *c-MET* pathway may prevent disease progression in NMIBC patients and improve the survival of MIBC patients. Also, multi-combination or co-targeting therapies might be needed to prevent acquired drug resistance. Yeh et al. demonstrated that 21.5% (14/65) of patients co-expressing *c-MET*/*AXL*/*PDGFR* showed poor long-term survival (p = 0.015) [11]. However, they only identified a clinical correlation between *c-MET* network genes in patients with locally advanced and metastatic BCa. Here, we identified a clinical correlation in patients with NMIBC or MIBC. Thus, the present study suggests that the *c-MET* network is a promising biomarker and target for co-targeting drugs; this should be tested in clinical trials involving both NMIBC and MIBC patients.

Taken together, these data suggest that (1) *c-MET*/*AXL*/*PDGFR* levels can be used to distinguish cancer patients from normal controls and to distinguish NMIBC from MIBC; and (2) the expression of *c-MET* network genes is significantly associated with poorer survival rates for BCa patients.

Identifying the signaling networks involved may provide information that will further our understanding of the mechanisms underlying tumor biology and help to predict potential drug resistance [30]. We believe that *c-MET* network gene expression is a novel prognostic marker for predicting which BCa patients have an increased risk of developing aggressive disease. These genes might be a useful marker for co-targeting therapy, and are expected to play an important role in improving both response to treatment and survival of BCa patients.

## Supporting Information

S1 Table Dataset with recurrence, progression, and 5 genes in NMIBC patients. (PDF)

S2 Table Dataset with progression, overall survival, cancer specific survival, and 5 genes in MIBC patients. (PDF)

S3 Table Dataset with recurrence, progression, and *C-MET* network genes in NMIBC patients. (PDF)

S4 Table Dataset with progression, overall survival, cancer specific survival, and *C-MET* network genes in MIBC patients. (PDF)

## References

[1] Nguyen PL, Swanson PE, Jaszcz W, Aeppli DM, Zhang G, Singleton TP, et al Expression of epidermal growth factor receptor in invasive transitional cell carcinoma of the urinary bladder. A multivariate survival analysis. Am J Clin Pathol. 1994;101(2):166–76. . 7906919 10.1093/ajcp/101.2.166

[2] Tsai YS, Cheng HL, Tzai TS, Chow NH. Clinical Significance of ErbB Receptor Family in Urothelial Carcinoma of the Bladder: A Systematic Review and Meta-Analysis. Adv Urol. 2008;2012:181964 10.1155/2012/181964 . 22991510 PMC3443987

[3] Ciardiello F, Tortora G. EGFR antagonists in cancer treatment. N Engl J Med. 2008;358(11):1160–74. 10.1056/NEJMra0707704 . 18337605

[4] Noon AP, Catto JW. Bladder cancer in 2012: Challenging current paradigms. Nat Rev Urol. 2013;10(2):67–8. 10.1038/nrurol.2012.252 . 23295237

[5] Cheng HL, Trink B, Tzai TS, Liu HS, Chan SH, Ho CL, et al Overexpression of c-met as a prognostic indicator for transitional cell carcinoma of the urinary bladder: a comparison with p53 nuclear accumulation. J Clin Oncol. 2002;20(6):1544–50. . 11896103 10.1200/JCO.2002.20.6.1544

[6] Peters S, Adjei AA. MET: a promising anticancer therapeutic target. Nat Rev Clin Oncol. 2012;9(6):314–26. 10.1038/nrclinonc.2012.71 . 22566105

[7] Miyata Y, Sagara Y, Kanda S, Hayashi T, Kanetake H. Phosphorylated hepatocyte growth factor receptor/c-Met is associated with tumor growth and prognosis in patients with bladder cancer: correlation with matrix metalloproteinase-2 and -7 and E-cadherin. Hum Pathol. 2009;40(4):496–504. 10.1016/j.humpath.2008.09.011 . 19121849

[8] Er EE, Mendoza MC, Mackey AM, Rameh LE, Blenis J. AKT facilitates EGFR trafficking and degradation by phosphorylating and activating PIKfyve. Sci Signal. 2013;6(279):ra45 10.1126/scisignal.2004015 . 23757022 PMC4041878

[9] Gherardi E, Birchmeier W, Birchmeier C, Vande Woude G. Targeting MET in cancer: rationale and progress. Nat Rev Cancer. 2012;12(2):89–103. 10.1038/nrc3205 . 22270953

[10] Xu X, Chen H, Lin Y, Hu Z, Mao Y, Wu J, et al MicroRNA-409-3p inhibits migration and invasion of bladder cancer cells via targeting c-Met. Mol Cells. 2013;36(1):62–8. 10.1007/s10059-013-0044-7 . 23820886 PMC3887926

[11] Yeh CY, Shin SM, Yeh HH, Wu TJ, Shin JW, Chang TY, et al Transcriptional activation of the Axl and PDGFR-alpha by c-Met through a ras- and Src-independent mechanism in human bladder cancer. BMC Cancer. 2011;11:139 10.1186/1471-2407-11-139 . 21496277 PMC3101176

[12] Hofner T, Macher-Goeppinger S, Klein C, Rigo-Watermeier T, Eisen C, Pahernik S, et al Development and characteristics of preclinical experimental models for the research of rare neuroendocrine bladder cancer. J Urol. 2013;190(6):2263–70. 10.1016/j.juro.2013.06.053 . 23820058

[13] Comperat E, Roupret M, Chartier-Kastler E, Bitker MO, Richard F, Camparo P, et al Prognostic value of MET, RON and histoprognostic factors for urothelial carcinoma in the upper urinary tract. J Urol. 2008;179(3):868–72; discussion 72. 10.1016/j.juro.2007.10.079 . 18221954

[14] Carvalho I, Milanezi F, Martins A, Reis RM, Schmitt F. Overexpression of platelet-derived growth factor receptor alpha in breast cancer is associated with tumour progression. Breast Cancer Res. 2005;7(5):R788–95. 10.1186/bcr1304 ; PMCID: PMC1242156. 16168125 PMC1242156

[15] Chung BI, Malkowicz SB, Nguyen TB, Libertino JA, McGarvey TW. Expression of the proto-oncogene Axl in renal cell carcinoma. DNA Cell Biol. 2003;22(8):533–40. 10.1089/10445490360708946 . 14565870

[16] Shieh YS, Lai CY, Kao YR, Shiah SG, Chu YW, Lee HS, et al Expression of axl in lung adenocarcinoma and correlation with tumor progression. Neoplasia. 2005;7(12):1058–64. ; PMCID: PMC1501169. 16354588 10.1593/neo.05640 PMC1501169

[17] Donnem T, Al-Saad S, Al-Shibli K, Andersen S, Busund LT, Bremnes RM. Prognostic impact of platelet-derived growth factors in non-small cell lung cancer tumor and stromal cells. J Thorac Oncol. 2008;3(9):963–70. 10.1097/JTO.0b013e3181834f52 . 18758297

[18] Sainaghi PP, Castello L, Bergamasco L, Galletti M, Bellosta P, Avanzi GC. Gas6 induces proliferation in prostate carcinoma cell lines expressing the Axl receptor. J Cell Physiol. 2005;204(1):36–44. 10.1002/jcp.20265 . 15605394

[19] Fudge K, Bostwick DG, Stearns ME. Platelet-derived growth factor A and B chains and the alpha and beta receptors in prostatic intraepithelial neoplasia. Prostate. 1996;29(5):282–6. . 8899000 10.1002/(SICI)1097-0045(199611)29:5<282::AID-PROS2>3.0.CO;2-C

[20] Babjuk M, Oosterlinck W, Sylvester R, Kaasinen E, Bohle A, Palou-Redorta J. EAU guidelines on non-muscle-invasive urothelial carcinoma of the bladder. Eur Urol. 2008;54(2):303–14. 10.1016/j.eururo.2008.04.051 . 18468779

[21] Greene FL. The American Joint Committee on Cancer: updating the strategies in cancer staging. Bull Am Coll Surg. 2002;87(7):13–5. . 17387902

[22] Kim J, Ji M, DiDonato JA, Rackley RR, Kuang M, Sadhukhan PC, et al An hTERT-immortalized human urothelial cell line that responds to anti-proliferative factor. In Vitro Cell Dev Biol Anim. 2011;47(1):2–9. 10.1007/s11626-010-9350-y . 21136194 PMC3029472

[23] Kim J, Adam RM, Freeman MR. Trafficking of nuclear heparin-binding epidermal growth factor-like growth factor into an epidermal growth factor receptor-dependent autocrine loop in response to oxidative stress. Cancer Res. 2005;65(18):8242–9. 10.1158/0008-5472.CAN-05-0942 . 16166300

[24] Scagliotti GV, Novello S, von Pawel J. The emerging role of MET/HGF inhibitors in oncology. Cancer Treat Rev. 2013;39(7):793–801. 10.1016/j.ctrv.2013.02.001 . 23453860

[25] Hazan RB, Phillips GR, Qiao RF, Norton L, Aaronson SA. Exogenous expression of N-cadherin in breast cancer cells induces cell migration, invasion, and metastasis. J Cell Biol. 2000;148(4):779–90. . 10684258 10.1083/jcb.148.4.779 PMC2169367

[26] Van Lint P, Libert C. Chemokine and cytokine processing by matrix metalloproteinases and its effect on leukocyte migration and inflammation. J Leukoc Biol. 2007;82(6):1375–81. 10.1189/jlb.0607338 . 17709402

[27] Arena S, Pisacane A, Mazzone M, Comoglio PM, Bardelli A. Genetic targeting of the kinase activity of the Met receptor in cancer cells. Proc Natl Acad Sci U S A. 2007;104(27):11412–7. 10.1073/pnas.0703205104 . 17595299 PMC2040912

[28] Smith DC, Smith MR, Sweeney C, Elfiky AA, Logothetis C, Corn PG, et al Cabozantinib in patients with advanced prostate cancer: results of a phase II randomized discontinuation trial. J Clin Oncol. 2012;31(4):412–9. 10.1200/JCO.2012.45.0494 . 23169517 PMC4110249

[29] Remon J, Moran T, Majem M, Reguart N, Dalmau E, Marquez-Medina D, et al Acquired resistance to epidermal growth factor receptor tyrosine kinase inhibitors in EGFR-mutant non-small cell lung cancer: a new era begins. Cancer Treat Rev. 2013;40(1):93–101. 10.1016/j.ctrv.2013.06.002 . 23829935

[30] Rikova K, Guo A, Zeng Q, Possemato A, Yu J, Haack H, et al Global survey of phosphotyrosine signaling identifies oncogenic kinases in lung cancer. Cell. 2007;131(6):1190–203. 10.1016/j.cell.2007.11.025 . 18083107

