# Supplementary material for: The c-MET Network as Novel Prognostic Marker for Predicting Bladder Cancer Patients with an Increased Risk of Developing Aggressive Disease
Source: PLoS One. 2015 Jul 30;10(7):e0134552. doi: 10.1371/journal.pone.0134552 (PMC4520492; doi:10.1371/journal.pone.0134552)
Supplement: S1 Table — (PDF) [file pone.0134552.s001.pdf]

NMIBC _ each gene										
	PDGFRB	MET	AXL	recur month	recur-rence	prog-ression month	prog-ression	PDGFRL	PDGFRB	PDGFRA
BT001.SUP	7.62	10.61	9.37	4.13	1.00	18.17	0.00	7.21	7.62	7.17
BT002.SUP	7.69	11.42	9.82	173.50	0.00	173.50	0.00	7.14	7.69	7.19
BT003.SUP	9.67	10.65	10.00	172.90	0.00	172.90	0.00	8.31	9.67	8.57
BT004.SUP	8.24	9.75	8.12	26.90	0.00	26.90	0.00	7.31	8.24	7.03
BT005.SUP	9.6	11.31	7.77	87.07	0.00	87.07	0.00	7.25	9.6	7.2
BT006.SUP	9.23	9.84	9.36	15.30	0.00	15.30	0.00	7.25	9.23	7.64
BT007.SUP	9.4	9.65	8.43	4.07	1.00	17.17	1.00	7.08	9.4	7.96
BT011.SUP	9.14	9.74	9.09	161.57	0.00	161.57	0.00	7.49	9.14	7.59
BT012.SUP	9.33	10.50	10.15	161.80	0.00	161.80	0.00	7.47	9.33	7.7
BT013.SUP	9.18	9.78	9.41	167.13	0.00	167.13	0.00	7.61	9.18	7.76
BT015.SUP	10.08	9.62	8.58	132.27	0.00	132.27	0.00	7.02	10.08	6.99
BT017.SUP	8.89	10.20	8.12	9.80	1.00	164.17	0.00	7.29	8.89	7.69
BT018.SUP	9.75	9.51	8.60	17.53	1.00	69.10	1.00	8.2	9.75	7.72
BT020.SUP	8.79	9.90	9.06	65.93	0.00	65.93	0.00	7.49	8.79	7.98
BT021.SUP	9.04	9.58	9.08	3.23	0.00	3.23	0.00	7.18	9.04	7.66
BT022.SUP	9.96	9.68	9.25	157.13	0.00	157.13	0.00	7.12	9.96	7.67
BT023.SUP	10.83	10.10	9.46	3.43	1.00	22.67	0.00	7.23	10.83	6.98
BT025.SUP	11.28	10.17	9.71	50.43	0.00	50.43	0.00	7.39	11.28	8.87
BT027.SUP	8.8	10.45	8.47	156.70	0.00	156.70	0.00	7.37	8.8	7.27
BT028.SUP	9.46	10.16	9.00	6.57	1.00	157.13	0.00	7.19	9.46	8.81
BT031.SUP	11.23	10.17	9.30	97.63	1.00	143.13	0.00	7.03	11.23	9.12
BT033.SUP	8.62	10.00	9.23	98.00	0.00	98.00	0.00	7.27	8.62	7.91
BT034.SUP	9.93	9.80	9.18	27.53	1.00	66.97	1.00	7.06	9.93	6.94
BT035.SUP	9.67	9.75	9.55	104.03	0.00	104.03	0.00	7.55	9.67	9.4
BT036.SUP	9.33	10.39	9.12	18.60	1.00	18.60	1.00	8.33	9.33	8.08
BT037.SUP	9.35	8.47	7.97	134.77	0.00	134.77	0.00	7.54	9.35	7.44
BT038.SUP	10.34	10.99	9.24	9.50	1.00	142.57	0.00	7.53	10.34	9.73
BT039.SUP	9.59	9.91	8.69	131.83	0.00	131.83	0.00	7.36	9.59	7.1
BT040.SUP	9.41	10.17	8.79	80.43	0.00	80.43	0.00	7.16	9.41	7.29
BT041.SUP	9.4	10.54	8.88	6.63	1.00	6.63	1.00	7.28	9.4	8.07
BT042.SUP	10.22	10.48	8.49	13.67	1.00	51.77	0.00	8.58	10.22	8.09
BT043.SUP	9.1	9.89	8.22	129.37	0.00	129.37	0.00	7.41	9.1	7
BT045.SUP	9.54	10.63	9.79	127.00	0.00	127.00	0.00	7.66	9.54	8.62
BT046.SUP	11.24	9.84	9.49	122.60	0.00	122.60	0.00	7	11.24	9.52
BT047.SUP	10.71	10.21	9.25	6.30	1.00	108.30	1.00	7.75	10.71	8.27
BT048.SUP	9.88	9.75	9.06	12.83	1.00	65.93	0.00	7.64	9.88	7.33
BT051.SUP	10.67	9.11	8.83	85.43	0.00	85.43	0.00	8.32	10.67	8.16
BT052.SUP	9.88	10.05	8.41	3.17	0.00	3.17	0.00	7.7	9.88	8.53
BT053.SUP	10.28	10.01	9.60	4.00	1.00	128.47	0.00	7.7	10.28	7.93

BT054.SUP	10.64	9.12	9.18	120.17	0.00	120.17	0.00	7.1	10.64	8.69
BT055.SUP	9.24	10.21	9.29	26.07	0.00	26.07	0.00	8	9.24	8.73
BT057.SUP	9.56	10.76	9.40	64.43	1.00	116.73	0.00	7.78	9.56	7.89
BT058.SUP	10.58	10.02	8.27	65.30	1.00	115.37	1.00	8.39	10.58	8.43
BT059.SUP	9.98	10.58	9.10	115.27	0.00	115.27	0.00	8.21	9.98	8.98
BT060.SUP	9.78	9.43	8.93	50.87	1.00	50.87	1.00	9.06	9.78	7.7
BT062.SUP	9.74	9.67	8.40	17.23	1.00	58.13	1.00	7.61	9.74	7.15
BT063.SUP	8.88	9.79	8.21	32.33	1.00	112.23	0.00	6.99	8.88	7.68
BT064.SUP	8.24	9.16	8.09	111.67	0.00	111.67	0.00	7.14	8.24	7.62
BT065.SUP	8.95	11.08	8.27	68.90	1.00	111.67	0.00	7.25	8.95	8.74
BT066.SUP	9.2	8.89	10.28	110.70	0.00	110.70	0.00	7.25	9.2	10.23
BT067.SUP	10.63	9.66	9.50	9.80	1.00	16.23	0.00	7.2	10.63	7.24
BT071.SUP	10.06	9.63	8.87	16.57	1.00	25.03	0.00	8.35	10.06	7.05
BT072.SUP	10.23	8.93	9.85	103.60	0.00	103.60	0.00	7.92	10.23	9.9
BT075.SUP	9.87	9.55	8.55	97.73	1.00	98.53	0.00	8.91	9.87	8.5
BT076.SUP	9.53	11.71	8.60	97.87	0.00	97.87	0.00	7.18	9.53	7.18
BT077.SUP	10.09	9.06	9.26	89.47	1.00	97.43	0.00	7.66	10.09	7.92
BT078.SUP	9.22	10.18	9.55	96.73	0.00	96.73	0.00	7.3	9.22	7.89
BT079.SUP	10.06	9.04	8.97	96.13	0.00	96.13	0.00	7.38	10.06	8.92
BT081.SUP	9.94	9.49	9.35	59.87	0.00	59.87	0.00	7.27	9.94	7.25
BT082.SUP	9.95	10.78	9.84	18.43	1.00	95.53	0.00	7.64	9.95	8.91
BT083.SUP	12.28	9.30	11.34	14.53	1.00	95.27	0.00	8.1	12.28	8.63
BT084.SUP	10.44	9.23	8.91	46.17	0.00	46.17	0.00	7.16	10.44	7.53
BT085.SUP	10.33	8.54	9.32	94.97	0.00	94.97	0.00	9.47	10.33	8.7
BT086.SUP	11.38	9.25	9.97	94.60	0.00	94.60	0.00	7.39	11.38	8.02
BT087.SUP	10.27	10.07	10.54	6.07	1.00	94.33	0.00	8.21	10.27	7.16
BT088.SUP	9.97	10.23	8.13	36.30	0.00	36.30	0.00	7.13	9.97	7.44
BT091.SUP	8.99	8.75	9.92	89.50	0.00	89.50	0.00	7.23	8.99	9.11
BT094.SUP	9.61	10.17	8.18	7.23	1.00	27.03	1.00	8.43	9.61	8.44
BT095.SUP	12.28	8.28	10.82	12.17	1.00	12.17	1.00	7.98	12.28	9.72
BT099.SUP	9.54	9.55	9.41	3.17	1.00	84.00	0.00	7.72	9.54	8.86
BT103.SUP	9.04	10.24	8.65	4.90	1.00	80.03	0.00	7.37	9.04	7.29
BT104.SUP	10.24	10.33	8.93	80.63	0.00	80.63	0.00	8.56	10.24	7.9
BT106.SUP	10.99	9.63	9.40	31.53	0.00	31.53	0.00	9.35	10.99	8.67
BT107.SUP	10.25	11.02	8.69	78.33	0.00	67.77	1.00	7.37	10.25	7.91
BT109.SUP	12.28	9.83	10.21	9.10	1.00	77.60	0.00	7.15	12.28	10.14
BT113.SUP	10.43	10.89	8.52	31.97	0.00	31.97	0.00	7.67	10.43	7.97
BT114.SUP	9.23	9.81	8.98	73.83	0.00	73.83	0.00	7.43	9.23	7.98
BT116.SUP	10.85	9.29	10.31	73.10	0.00	73.10	0.00	7.39	10.85	9.04
BT119.SUP	11.43	8.56	9.87	23.53	0.00	23.53	0.00	8.01	11.43	9.32
BT121.SUP	9.3	9.63	10.01	71.40	0.00	71.40	0.00	7.12	9.3	9.51
BT123.SUP	9.82	10.36	9.05	70.23	0.00	70.23	0.00	7.26	9.82	9.11
BT127.SUP	10.07	10.53	9.69	69.40	0.00	69.40	0.00	7.37	10.07	9.81

BT131.SUP	10.52	10.13	8.88	23.57	0.00	23.57	0.00	7.62	10.52	7.82
BT132.SUP	9.34	10.32	8.48	15.43	1.00	65.47	0.00	7.28	9.34	8.96
BT133.SUP	10.69	8.04	9.46	35.13	0.00	35.13	0.00	7.25	10.69	8.11
BT134.SUP	10.07	10.66	8.76	20.77	1.00	66.27	0.00	7.18	10.07	8.28
BT135.SUP	9.38	10.62	9.18	15.43	0.00	15.43	0.00	7.34	9.38	8.15
BT136.SUP	11	9.40	10.15	65.90	0.00	65.90	0.00	7.37	11	8.55
BT137.SUP	10.33	10.14	9.91	17.67	0.00	17.67	0.00	7.82	10.33	10.23
BT138.SUP	10.9	9.73	9.61	65.00	0.00	65.00	0.00	7.12	10.9	6.97
BT139.SUP	11.91	10.45	11.07	3.00	1.00	60.43	0.00	7.22	11.91	8.92
BT140.SUP	11.09	10.81	9.68	60.10	0.00	60.10	0.00	8.14	11.09	8.5
BT141.SUP	11.23	9.25	8.93	3.27	1.00	13.93	0.00	7.46	11.23	9.72
BT145.SUP	8.42	10.47	9.17	3.47	1.00	58.20	0.00	7.28	8.42	8.27
BT146.SUP	11.29	9.79	10.97	13.63	0.00	13.63	0.00	8.06	11.29	11.8
BT147.SUP	11.86	8.72	10.95	57.93	0.00	57.93	0.00	7.97	11.86	8.66
BT148.SUP	11.01	9.84	9.38	57.87	0.00	57.87	0.00	8.29	11.01	9.67
BT149.SUP	11.68	10.11	10.06	27.50	0.00	14.97	1.00	7.56	11.68	9.68
BT150.SUP	10.56	10.64	8.91	57.77	0.00	57.77	0.00	7.09	10.56	7.8
BT151.SUP	9.86	9.95	9.31	11.97	1.00	57.00	0.00	7.8	9.86	9.27
BT152.SUP	10.86	9.92	9.12	56.93	0.00	56.93	0.00	9.1	10.86	9.59
BT153.SUP	9.77	8.53	9.30	8.33	1.00	55.53	0.00	7.41	9.77	8.99
BT165.SUP	11.25	8.57	9.02	43.03	0.00	43.03	0.00	7.58	11.25	7.72
