# Supplementary material for: The c-MET Network as Novel Prognostic Marker for Predicting Bladder Cancer Patients with an Increased Risk of Developing Aggressive Disease
Source: PLoS One. 2015 Jul 30;10(7):e0134552. doi: 10.1371/journal.pone.0134552 (PMC4520492; doi:10.1371/journal.pone.0134552)
Supplement: S2 Table — (PDF) [file pone.0134552.s002.pdf]

MIBC_Each gene											
	PDGFRB	MET	AXL	prog-ression month	prog-ression	survival Month	cancer-specific survival	overall survival	PDGFRL	PDGFRB	PDGFRA
BT008.INV	11.74	9.23	11.17	166.47	0.00	166.47	0.00	0.00	8.12	11.74	9.09
BT009.INV	9.37	9.48	10.02	12.57	0.00	12.57	1.00	1.00	8.55	9.37	7.09
BT010.INV	10.38	9.10	8.56	165.50	0.00	165.50	0.00	0.00	7.61	10.38	8.06
BT014.INV	9.11	9.43	8.24	1.03	0.00	1.03	1.00	1.00	7.3	9.11	8.39
BT016.INV	11.07	9.04	9.99	16.67	0.00	16.67	1.00	1.00	7.22	11.07	8.28
BT019.INV	10.97	9.14	8.36	3.13	0.00	3.13	1.00	1.00	7.18	10.97	7.1
BT024.INV	10.44	9.86	9.70	106.83	1.00	157.03	0.00	0.00	7.64	10.44	7.3
BT026.INV	10.00	10.18	9.18	53.77	1.00	66.30	1.00	1.00	7.17	10	7.69
BT029.INV	10.01	8.86	8.79	8.93	1.00	14.57	1.00	1.00	7.53	10.01	8.03
BT030.INV	10.00	10.34	8.70	152.37	0.00	152.37	0.00	0.00	7.33	10	8.33
BT032.INV	8.26	9.03	8.18	157.73	0.00	157.73	0.00	0.00	8.19	8.26	7
BT044.INV	10.03	10.48	9.11	127.27	0.00	127.27	0.00	0.00	7.34	10.03	7.88
BT049.INV	10.42	9.16	9.41	25.77	1.00	25.83	1.00	1.00	8.39	10.42	8.46
BT050.INV	11.12	10.09	9.95	10.27	0.00	10.27	1.00	1.00	9.72	11.12	8.08
BT056.INV	11.80	8.24	11.75	118.20	0.00	118.17	0.00	0.00	9.08	11.8	8.76
BT061.INV	11.85	9.51	9.43	8.70	0.00	8.70	1.00	1.00	7.8	11.85	8.98
BT068.INV	11.11	9.77	9.61	3.77	1.00	15.10	1.00	1.00	7.14	11.11	8.89
BT069.INV	10.76	10.23	8.30	2.80	1.00	11.23	1.00	1.00	9.16	10.76	7.09
BT070.INV	11.68	8.34	11.56	105.97	0.00	105.97	0.00	0.00	9.13	11.68	9.82
BT073.INV	9.47	10.19	8.73	101.77	0.00	101.77	0.00	0.00	7.99	9.47	8.58
BT074.INV	11.03	9.75	10.41	11.97	0.00	11.97	1.00	1.00	9.29	11.03	8.08
BT080.INV	10.85	10.32	10.70	7.30	1.00	10.40	1.00	1.00	7.11	10.85	8.52
BT089.INV	10.70	9.01	9.73	12.97	1.00	93.00	0.00	0.00	7.64	10.7	8.01
BT090.INV	9.90	9.19	9.34	60.73	1.00	81.87	1.00	1.00	7.76	9.9	9.19
BT092.INV	11.02	11.10	10.35	3.60	1.00	11.50	1.00	1.00	7.63	11.02	8.67
BT093.INV	12.82	8.61	11.20	15.37	1.00	17.87	0.00	1.00	8.83	12.82	11.15
BT096.INV	9.84	9.07	8.40	6.63	1.00	15.40	1.00	1.00	7.8	9.84	11.68
BT097.INV	9.78	8.79	8.29	84.97	0.00	84.97	0.00	0.00	9.22	9.78	11.32
BT098.INV	11.28	9.97	9.59	4.50	0.00	4.50	1.00	1.00	7.5	11.28	8.93
BT100.INV	9.50	8.69	8.09	5.23	0.00	5.23	1.00	1.00	8.44	9.5	9
BT101.INV	12.91	9.32	11.51	82.13	0.00	82.13	0.00	0.00	7.77	12.91	8.03
BT102.INV	10.10	9.42	9.07	81.90	0.00	81.67	0.00	0.00	7.25	10.1	7.5
BT105.INV	11.30	12.53	10.97	5.93	0.00	5.93	0.00	1.00	9.52	11.3	7.78
BT108.INV	10.80	9.24	9.25	6.50	0.00	6.50	1.00	1.00	7.35	10.8	9.53
BT110.INV	11.48	9.26	11.61	76.30	0.00	76.30	0.00	0.00	7.45	11.48	7.64

BT111.INV	10.33	9.68	9.03	75.50	0.00	75.50	0.00	0.00	7.28	10.33	8.71
BT115.INV	11.56	8.84	10.49	35.80	0.00	36.83	0.00	1.00	7.36	11.56	9.48
BT117.INV	12.71	9.20	11.04	25.87	1.00	73.03	0.00	0.00	8.54	12.71	8.4
BT118.INV	10.88	8.64	9.43	3.57	1.00	3.93	1.00	1.00	8.72	10.88	9.18
BT120.INV	10.36	9.20	9.08	13.07	1.00	15.10	1.00	1.00	8.03	10.36	8.36
BT122.INV	11.74	9.73	9.79	47.20	0.00	47.20	0.00	1.00	8.41	11.74	8.91
BT124.INV	10.85	10.20	10.38	10.90	1.00	11.07	1.00	1.00	7.55	10.85	9.13
BT125.INV	9.82	10.17	9.99	3.53	1.00	5.77	1.00	1.00	7.51	9.82	7.2
BT126.INV	9.51	9.83	7.77	69.60	0.00	69.60	0.00	0.00	9.67	9.51	7.44
BT128.INV	10.01	11.65	9.62	9.23	0.00	9.23	0.00	1.00	7.44	10.01	7.43
BT129.INV	10.27	11.15	12.26	3.27	1.00	7.10	1.00	1.00	8.62	10.27	8.67
BT130.INV	11.30	10.41	11.35	17.13	0.00	17.13	0.00	1.00	7.64	11.3	8.75
BT142.INV	9.65	8.84	9.26	2.13	0.00	2.13	1.00	1.00	7.7	9.65	7.58
BT143.INV	13.46	8.41	11.87	58.80	0.00	58.80	0.00	0.00	10.17	13.46	9.46
BT144.INV	9.61	10.63	9.11	6.87	0.00	6.87	0.00	1.00	7.63	9.61	7.99
BT154.INV	9.30	9.53	8.63	20.73	1.00	115.83	0.00	0.00	9.09	9.3	7.48
BT155.INV	12.59	9.53	10.27	8.13	1.00	15.47	1.00	1.00	8.74	12.59	9.14
BT156.INV	12.19	9.02	11.72	26.43	0.00	26.43	1.00	1.00	9.09	12.19	10.07
BT157.INV	11.35	9.70	9.19	13.27	0.00	13.27	1.00	1.00	9.18	11.35	8.16
BT158.INV	12.89	12.30	11.71	1.17	1.00	6.40	1.00	1.00	10.29	12.89	10.65
BT159.INV	10.31	10.41	9.49	10.67	0.00	10.67	1.00	1.00	7.52	10.31	9.49
BT160.INV	11.41	10.22	9.68	54.67	0.00	54.67	0.00	0.00	7.47	11.41	9.01
BT161.INV	11.01	9.48	9.79	51.90	0.00	51.90	0.00	0.00	7.28	11.01	8.31
BT162.INV	10.22	10.41	9.43	16.80	1.00	18.47	1.00	1.00	8.06	10.22	8.07
BT163.INV	9.27	10.71	12.92	1.37	0.00	1.37	0.00	1.00	7.91	9.27	7.6
BT164.INV	10.25	9.38	9.26	9.00	1.00	46.57	0.00	0.00	8.43	10.25	7.45
BT166.INV	12.83	10.60	10.69	41.83	0.00	41.83	0.00	0.00	8.68	12.83	11
