# Supplementary material for: The c-MET Network as Novel Prognostic Marker for Predicting Bladder Cancer Patients with an Increased Risk of Developing Aggressive Disease
Source: PLoS One. 2015 Jul 30;10(7):e0134552. doi: 10.1371/journal.pone.0134552 (PMC4520492; doi:10.1371/journal.pone.0134552)
Supplement: S3 Table — (PDF) [file pone.0134552.s003.pdf]

C-Met network NMIBC						
	recur month	recurrence	class_gene _Rec	progression month	progression	class_gene _Prog
BT001.SUP	4.13	1.00	1.00	18.17	0.00	1.00
BT002.SUP	173.50	0.00	1.00	173.50	0.00	1.00
BT003.SUP	172.90	0.00	2.00	172.90	0.00	1.00
BT004.SUP	26.90	0.00	1.00	26.90	0.00	1.00
BT005.SUP	87.07	0.00	2.00	87.07	0.00	2.00
BT006.SUP	15.30	0.00	1.00	15.30	0.00	1.00
BT007.SUP	4.07	1.00	1.00	17.17	1.00	2.00
BT011.SUP	161.57	0.00	1.00	161.57	0.00	1.00
BT012.SUP	161.80	0.00	1.00	161.80	0.00	1.00
BT013.SUP	167.13	0.00	1.00	167.13	0.00	1.00
BT015.SUP	132.27	0.00	2.00	132.27	0.00	2.00
BT017.SUP	9.80	1.00	2.00	164.17	0.00	2.00
BT018.SUP	17.53	1.00	1.00	69.10	1.00	2.00
BT020.SUP	65.93	0.00	1.00	65.93	0.00	1.00
BT021.SUP	3.23	0.00	1.00	3.23	0.00	1.00
BT022.SUP	157.13	0.00	1.00	157.13	0.00	1.00
BT023.SUP	3.43	1.00	2.00	22.67	0.00	2.00
BT025.SUP	50.43	0.00	2.00	50.43	0.00	2.00
BT027.SUP	156.70	0.00	2.00	156.70	0.00	1.00
BT028.SUP	6.57	1.00	2.00	157.13	0.00	1.00
BT031.SUP	97.63	1.00	2.00	143.13	0.00	2.00
BT033.SUP	98.00	0.00	1.00	98.00	0.00	1.00
BT034.SUP	27.53	1.00	1.00	66.97	1.00	1.00
BT035.SUP	104.03	0.00	1.00	104.03	0.00	1.00
BT036.SUP	18.60	1.00	2.00	18.60	1.00	1.00
BT037.SUP	134.77	0.00	1.00	134.77	0.00	2.00
BT038.SUP	9.50	1.00	2.00	142.57	0.00	1.00
BT039.SUP	131.83	0.00	2.00	131.83	0.00	2.00
BT040.SUP	80.43	0.00	2.00	80.43	0.00	1.00
BT041.SUP	6.63	1.00	2.00	6.63	1.00	1.00
BT042.SUP	13.67	1.00	2.00	51.77	0.00	2.00
BT043.SUP	129.37	0.00	1.00	129.37	0.00	2.00
BT045.SUP	127.00	0.00	2.00	127.00	0.00	1.00
BT046.SUP	122.60	0.00	2.00	122.60	0.00	2.00
BT047.SUP	6.30	1.00	2.00	108.30	1.00	2.00
BT048.SUP	12.83	1.00	1.00	65.93	0.00	2.00
BT051.SUP	85.43	0.00	1.00	85.43	0.00	2.00
BT052.SUP	3.17	0.00	2.00	3.17	0.00	2.00
BT053.SUP	4.00	1.00	2.00	128.47	0.00	1.00
BT054.SUP	120.17	0.00	1.00	120.17	0.00	2.00

BT055.SUP	26.07	0.00	1.00	26.07	0.00	1.00
BT057.SUP	64.43	1.00	2.00	116.73	0.00	1.00
BT058.SUP	65.30	1.00	2.00	115.37	1.00	2.00
BT059.SUP	115.27	0.00	2.00	115.27	0.00	1.00
BT060.SUP	50.87	1.00	1.00	50.87	1.00	2.00
BT062.SUP	17.23	1.00	2.00	58.13	1.00	2.00
BT063.SUP	32.33	1.00	1.00	112.23	0.00	2.00
BT064.SUP	111.67	0.00	1.00	111.67	0.00	1.00
BT065.SUP	68.90	1.00	2.00	111.67	0.00	1.00
BT066.SUP	110.70	0.00	1.00	110.70	0.00	1.00
BT067.SUP	9.80	1.00	2.00	16.23	0.00	2.00
BT071.SUP	16.57	1.00	1.00	25.03	0.00	2.00
BT072.SUP	103.60	0.00	1.00	103.60	0.00	1.00
BT075.SUP	97.73	1.00	1.00	98.53	0.00	2.00
BT076.SUP	97.87	0.00	2.00	97.87	0.00	1.00
BT077.SUP	89.47	1.00	1.00	97.43	0.00	2.00
BT078.SUP	96.73	0.00	1.00	96.73	0.00	1.00
BT079.SUP	96.13	0.00	1.00	96.13	0.00	2.00
BT081.SUP	59.87	0.00	1.00	59.87	0.00	1.00
BT082.SUP	18.43	1.00	2.00	95.53	0.00	1.00
BT083.SUP	14.53	1.00	1.00	95.27	0.00	1.00
BT084.SUP	46.17	0.00	1.00	46.17	0.00	2.00
BT085.SUP	94.97	0.00	1.00	94.97	0.00	2.00
BT086.SUP	94.60	0.00	1.00	94.60	0.00	2.00
BT087.SUP	6.07	1.00	1.00	94.33	0.00	1.00
BT088.SUP	36.30	0.00	2.00	36.30	0.00	2.00
BT091.SUP	89.50	0.00	1.00	89.50	0.00	1.00
BT094.SUP	7.23	1.00	2.00	27.03	1.00	2.00
BT095.SUP	12.17	1.00	1.00	12.17	1.00	2.00
BT099.SUP	3.17	1.00	1.00	84.00	0.00	1.00
BT103.SUP	4.90	1.00	2.00	80.03	0.00	1.00
BT104.SUP	80.63	0.00	2.00	80.63	0.00	2.00
BT106.SUP	31.53	0.00	2.00	31.53	0.00	2.00
BT107.SUP	78.33	0.00	2.00	67.77	1.00	2.00
BT109.SUP	9.10	1.00	2.00	77.60	0.00	2.00
BT113.SUP	31.97	0.00	2.00	31.97	0.00	2.00
BT114.SUP	73.83	0.00	1.00	73.83	0.00	1.00
BT116.SUP	73.10	0.00	1.00	73.10	0.00	1.00
BT119.SUP	23.53	0.00	1.00	23.53	0.00	2.00
BT121.SUP	71.40	0.00	1.00	71.40	0.00	1.00
BT123.SUP	70.23	0.00	2.00	70.23	0.00	1.00
BT127.SUP	69.40	0.00	2.00	69.40	0.00	1.00
BT131.SUP	23.57	0.00	2.00	23.57	0.00	2.00

BT132.SUP	15.43	1.00	2.00	65.47	0.00	2.00
BT133.SUP	35.13	0.00	1.00	35.13	0.00	2.00
BT134.SUP	20.77	1.00	2.00	66.27	0.00	2.00
BT135.SUP	15.43	0.00	2.00	15.43	0.00	1.00
BT136.SUP	65.90	0.00	1.00	65.90	0.00	1.00
BT137.SUP	17.67	0.00	2.00	17.67	0.00	1.00
BT138.SUP	65.00	0.00	2.00	65.00	0.00	2.00
BT139.SUP	3.00	1.00	2.00	60.43	0.00	1.00
BT140.SUP	60.10	0.00	2.00	60.10	0.00	2.00
BT141.SUP	3.27	1.00	2.00	13.93	0.00	2.00
BT145.SUP	3.47	1.00	1.00	58.20	0.00	1.00
BT146.SUP	13.63	0.00	1.00	13.63	0.00	1.00
BT147.SUP	57.93	0.00	1.00	57.93	0.00	1.00
BT148.SUP	57.87	0.00	2.00	57.87	0.00	2.00
BT149.SUP	27.50	0.00	2.00	14.97	1.00	2.00
BT150.SUP	57.77	0.00	2.00	57.77	0.00	2.00
BT151.SUP	11.97	1.00	1.00	57.00	0.00	1.00
BT152.SUP	56.93	0.00	2.00	56.93	0.00	2.00
BT153.SUP	8.33	1.00	1.00	55.53	0.00	1.00
BT165.SUP	43.03	0.00	1.00	43.03	0.00	2.00
