# Supplementary material for: The c-MET Network as Novel Prognostic Marker for Predicting Bladder Cancer Patients with an Increased Risk of Developing Aggressive Disease
Source: PLoS One. 2015 Jul 30;10(7):e0134552. doi: 10.1371/journal.pone.0134552 (PMC4520492; doi:10.1371/journal.pone.0134552)
Supplement: S4 Table — (PDF) [file pone.0134552.s004.pdf]

C-Met network MIBC								
	prog-ression month	prog-ression	class_gene_Prog	cancer-specific survival	class_gene_CSS	overall survival	class_gene_OS	survival Month
BT008.INV	166.47	0.00	1.00	0.00	1.00	0.00	1.00	166.47
BT009.INV	12.57	0.00	2.00	1.00	2.00	1.00	2.00	12.57
BT010.INV	165.50	0.00	1.00	0.00	1.00	0.00	1.00	165.50
BT014.INV	1.03	0.00	1.00	1.00	2.00	1.00	2.00	1.03
BT016.INV	16.67	0.00	1.00	1.00	1.00	1.00	1.00	16.67
BT019.INV	3.13	0.00	1.00	1.00	1.00	1.00	1.00	3.13
BT024.INV	106.83	1.00	2.00	0.00	2.00	0.00	2.00	157.03
BT026.INV	53.77	1.00	2.00	1.00	2.00	1.00	2.00	66.30
BT029.INV	8.93	1.00	1.00	1.00	1.00	1.00	1.00	14.57
BT030.INV	152.37	0.00	2.00	0.00	2.00	0.00	2.00	152.37
BT032.INV	157.73	0.00	1.00	0.00	2.00	0.00	1.00	157.73
BT044.INV	127.27	0.00	2.00	0.00	2.00	0.00	2.00	127.27
BT049.INV	25.77	1.00	1.00	1.00	1.00	1.00	1.00	25.83
BT050.INV	10.27	0.00	2.00	1.00	2.00	1.00	2.00	10.27
BT056.INV	118.20	0.00	1.00	0.00	1.00	0.00	1.00	118.17
BT061.INV	8.70	0.00	1.00	1.00	1.00	1.00	1.00	8.70
BT068.INV	3.77	1.00	2.00	1.00	1.00	1.00	2.00	15.10
BT069.INV	2.80	1.00	2.00	1.00	2.00	1.00	2.00	11.23
BT070.INV	105.97	0.00	1.00	0.00	1.00	0.00	1.00	105.97
BT073.INV	101.77	0.00	2.00	0.00	2.00	0.00	2.00	101.77
BT074.INV	11.97	0.00	2.00	1.00	1.00	1.00	2.00	11.97
BT080.INV	7.30	1.00	2.00	1.00	2.00	1.00	2.00	10.40
BT089.INV	12.97	1.00	1.00	0.00	1.00	0.00	1.00	93.00
BT090.INV	60.73	1.00	1.00	1.00	1.00	1.00	1.00	81.87
BT092.INV	3.60	1.00	2.00	1.00	2.00	1.00	2.00	11.50
BT093.INV	15.37	1.00	1.00	0.00	1.00	1.00	1.00	17.87
BT096.INV	6.63	1.00	1.00	1.00	2.00	1.00	1.00	15.40
BT097.INV	84.97	0.00	1.00	0.00	1.00	0.00	1.00	84.97
BT098.INV	4.50	0.00	2.00	1.00	2.00	1.00	2.00	4.50
BT100.INV	5.23	0.00	1.00	1.00	2.00	1.00	1.00	5.23
BT101.INV	82.13	0.00	1.00	0.00	1.00	0.00	1.00	82.13
BT102.INV	81.90	0.00	1.00	0.00	2.00	0.00	1.00	81.67
BT105.INV	5.93	0.00	2.00	0.00	2.00	1.00	2.00	5.93
BT108.INV	6.50	0.00	1.00	1.00	1.00	1.00	1.00	6.50
BT110.INV	76.30	0.00	1.00	0.00	1.00	0.00	1.00	76.30
BT111.INV	75.50	0.00	2.00	0.00	2.00	0.00	2.00	75.50
BT115.INV	35.80	0.00	1.00	0.00	1.00	1.00	1.00	36.83
BT117.INV	25.87	1.00	1.00	0.00	1.00	0.00	1.00	73.03

BT118.INV	3.57	1.00	1.00	1.00	1.00	1.00	1.00	3.93
BT120.INV	13.07	1.00	1.00	1.00	1.00	1.00	1.00	15.10
BT122.INV	47.20	0.00	2.00	0.00	1.00	1.00	1.00	47.20
BT124.INV	10.90	1.00	2.00	1.00	2.00	1.00	2.00	11.07
BT125.INV	3.53	1.00	2.00	1.00	2.00	1.00	2.00	5.77
BT126.INV	69.60	0.00	2.00	0.00	2.00	0.00	2.00	69.60
BT128.INV	9.23	0.00	2.00	0.00	2.00	1.00	2.00	9.23
BT129.INV	3.27	1.00	2.00	1.00	2.00	1.00	2.00	7.10
BT130.INV	17.13	0.00	2.00	0.00	1.00	1.00	2.00	17.13
BT142.INV	2.13	0.00	1.00	1.00	1.00	1.00	1.00	2.13
BT143.INV	58.80	0.00	1.00	0.00	1.00	0.00	1.00	58.80
BT144.INV	6.87	0.00	2.00	0.00	2.00	1.00	2.00	6.87
BT154.INV	20.73	1.00	2.00	0.00	2.00	0.00	2.00	115.83
BT155.INV	8.13	1.00	1.00	1.00	1.00	1.00	1.00	15.47
BT156.INV	26.43	0.00	1.00	1.00	1.00	1.00	1.00	26.43
BT157.INV	13.27	0.00	2.00	1.00	1.00	1.00	2.00	13.27
BT158.INV	1.17	1.00	2.00	1.00	2.00	1.00	2.00	6.40
BT159.INV	10.67	0.00	2.00	1.00	2.00	1.00	2.00	10.67
BT160.INV	54.67	0.00	2.00	0.00	2.00	0.00	2.00	54.67
BT161.INV	51.90	0.00	1.00	0.00	1.00	0.00	1.00	51.90
BT162.INV	16.80	1.00	2.00	1.00	2.00	1.00	2.00	18.47
BT163.INV	1.37	0.00	2.00	0.00	2.00	1.00	2.00	1.37
BT164.INV	9.00	1.00	1.00	0.00	2.00	0.00	1.00	46.57
BT166.INV	41.83	0.00	2.00	0.00	1.00	0.00	2.00	41.83
